# Editorial for the Special Issue “Advances in Colloidal Hydrogels”

**DOI:** 10.3390/gels9050404

**Published:** 2023-05-11

**Authors:** Fuguo Liu, To Ngai

**Affiliations:** 1 College of Food Science and Engineering, Northwest A&F University, Xianyang 712100, China; fuguo@nwafu.edu.cn; 2 Department of Chemistry, The Chinese University of Hong Kong, Shatin, NT, Hong Kong, China

Hydrogels are three-dimensional polymer networks derived from hydrophilic macromonomers, which can be categorized as natural, synthetic, or hybrid hydrogels. They can be formed through physical and chemical crosslinking or a combination of both. Hydrogels have attracted growing attention from scientists due to their exceptional swelling capabilities, biocompatibility, and cell adhesion properties. This Special Issue of *Gels* highlights recent advancements in the field of novel food colloids and hydrogels, encompassing their preparation, modification techniques, formation mechanisms, structure, and phase behavior, along with applications in various sectors. The Special Issue features five articles addressing topics such as the influence of gummy dosage forms and sugar types on glycemic response management, walnut protein gel development, emulsion-filled gel formation mechanisms, and enhancement of resistant starch’s enzymolysis resistance, among others.

The five articles published in this Special Issue showcase the latest advancements in innovative colloid and hydrogel research. Gan et al. [1] explore the impact of different gummy varieties on glycemic response management, discovering that gummies can help reduce subjects’ glycemic responses and improve glucose homeostasis control. Lei et al. [2] examine the effect of NaCl concentration on the rheological, structural, and gelling properties of walnut protein isolate (WNPI)-κ-carrageenan (KC) composite gel, revealing that an appropriate concentration of Na+ (15 mM) significantly enhances the gel’s properties and offers technical support for its application in the food industry. Li et al. [3] create emulsion-filled gels using gelatin and whey protein isolate through heat-induced or enzyme-induced methods, comparing their rheology, texture properties, and microstructure, and finding distinct differences in macroscopic properties based on the preparation method. Liu et al. [4] investigate the effects of hydrothermal and microwave dual treatment and zein on the enzymolysis resistance of resistant starch (RS) type 2-high amylose corn starch (HACS), demonstrating that combining these treatments significantly increases the RS content in HACS, providing a promising strategy to enhance its resistance. Song et al. [5] develop zein nanocomposites using multifrequency ultrasound to promote the stability of high internal phase emulsions, observing that dual-frequency ultrasound-produced nanocomposites exhibit improved properties, such as smaller particle size, superior thermal stability, and increased storage stability.

In conclusion, this Special Issue offers valuable perspectives on the latest developments in colloidal hydrogels and their applications across various fields such as food, medicine, and agriculture. The articles emphasize the significance of colloid/hydrogel design and synthesis, in addition to their physical and chemical properties. The content of this Special Issue will serve as a helpful resource for researchers and scientists engaged in creating gel-based functional foods and pharmaceuticals.
